# Two new Rett syndrome families and review of the literature: expanding the knowledge of *MECP2 *frameshift mutations

**DOI:** 10.1186/1750-1172-6-58

**Published:** 2011-08-30

**Authors:** Kirstine Ravn, Gitte Roende, Morten Duno, Kathrine Fuglsang, Kristin L Eiklid, Zeynep Tümer, Jytte B Nielsen, Ola H Skjeldal

**Affiliations:** 1 Center for Rett syndrome, Kennedy Center, Glostrup, Denmark; 2 Center for Applied Human Molecular Genetics, Kennedy Center, Glostrup, Denmark; 3 Department of Clinical Genetics, University of Copenhagen, Rigshospitalet, Denmark; 4 Department of Medical Genetics, Oslo University Hospital HF, Ullevål Hospital, Oslo, Norway; 5 Women and Children's Clinic, Vestre Viken Hospital Thrust, Norway

## Abstract

**Background:**

Rett syndrome (RTT) is an X-linked dominant neurodevelopmental disorder, which is usually caused by *de novo *mutations in the *MECP2 *gene. More than 70% of the disease causing *MECP2 *mutations are eight recurrent C to T transitions, which almost exclusively arise on the paternally derived X chromosome. About 10% of the RTT cases have a C-terminal frameshift deletion in *MECP2*. Only few RTT families with a segregating *MECP2 *mutation, which affects female carriers with a phenotype of mental retardation or RTT, have been reported in the literature. In this study we describe two new RTT families with three and four individuals, respectively, and review the literature comparing the type of mutations and phenotypes observed in RTT families with those observed in sporadic cases. Based on these observations we also investigated origin of mutation segregation to further improve genetic counselling.

**Methods:**

*MECP2 *mutations were identified by direct sequencing. XCI studies were performed using the X-linked androgen receptor (*AR*) locus. The parental origin of *de novo MECP2 *frameshift mutations was investigated using intronic SNPs.

**Results:**

In both families a C-terminal frameshift mutation segregates. Clinical features of the mutation carriers vary from classical RTT to mild mental retardation. XCI profiles of the female carriers correlate to their respective geno-/phenotypes. The majority of the *de novo *frameshift mutations occur on the paternally derived X chromosome (7/9 cases), without a paternal age effect.

**Conclusions:**

The present study suggests a correlation between the intrafamilial phenotypic differences observed in RTT families and their respective XCI pattern in blood, in contrast to sporadic RTT cases where a similar correlation has not been demonstrated. Furthermore, we found *de novo MECP2 *frameshift mutations frequently to be of paternal origin, although not with the same high paternal occurrence as in sporadic cases with C to T transitions. This suggests further investigations of more families. This study emphasizes the need for thorough genetic counselling of families with a newly diagnosed RTT patient.

## Background

Rett syndrome (RTT; MIM# 312750) is an X-linked dominant neurodevelopmental disorder almost exclusively affecting females. The incidence is about 1 in 10.000 female births [1]. The first loss-of-function mutations in the methyl-CpG-binding protein 2 (*MECP2*; MIM# 300005) were reported in RTT patients in 1999 [2]. Since then, more than 200 different *MECP2 *mutations have been identified (RettBASE, http://mecp2.chw.edu.au). About 70% of reported RTT cases have one of the eight recurrent single basepair substitutions (missense or nonsense mutations), all of which are cytosine-to-thymine (C > T) transitions affecting the cytosine of the CpG dinucleotides. Small C-terminal deletions, with one or both breakpoints located within the "deleted prone region" (DPR) of exon 4, account for 10% of the cases [3]. The vast majority of RTT cases are sporadic (> 99%) and four studies have shown that the eight common C > T transition mutations arise almost exclusively on the paternally derived X chromosome in sporadic cases [4-7]. So far, the parental origin of *de novo *frameshift mutations has not been investigated in large cohorts of RTT cases. Hemizygous males with *de novo *or familial *MECP2 *mutations often have frameshift mutations caused either by single-nucleotide deletions/insertions or by small intragenic rearrangements [8,9].

The general knowledge regarding RTT families with affected females is sparse as only 10 families have been described in the literature [10-17]. Existence of such families are usually explained by germline mosaicism or skewed X chromosome inactivation (XCI) in the carrier mothers, who are either asymptomatic or have mild mental retardation (MR). While the eight recurrent C > T transitions mutations often result in classical or atypical RTT in sporadic cases, the C-terminal deletions lead to a broader spectrum of clinical features, from severe encephalopathy in hemizygous males to hemizygous females with classical RTT, mild MR and asymptomatic carriers [18-20].

In this study we describe two new unrelated families with frameshift mutations within the DPR, showing large intrafamilial clinical variability, ranging from classic RTT to mild MR. Furthermore, we compare the type of mutations observed in RTT families to those of sporadic cases and we present our study on the parental origin of *de novo MECP2 *frameshift mutations in our RTT cohort.

### Family A

**Figure 1:**
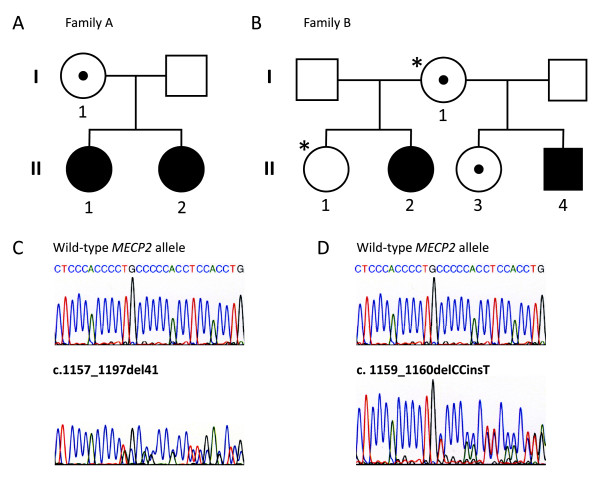
**Pedigree of the two families with RTT and the electrophoretograms of the *MECP2 *frameshift mutations**. (A) Family A, the carrier mother (I:1) and her two affected children, (B) Family B, the mother (I:1) who is an obligate carrier and her four children. * Family members who have not been tested for the *MECP2 *mutation in question. (C) Electrophoretograms showing the mutation, c.1157_1197del41, identified in family A (D) Electrophoretograms showing the mutation, c.1159_delCCinsT, identified in family B.

Family A, (Figure 1A), consists of a mother (patient I: 1) and her two daughters (patients II:1 and II:2).

Patient I:1 was married to a man with an IQ of 70 and have given birth to two affected children. The oldest daughter was removed to foster care at age 2.5 years, the youngest daughter immediately after birth. There is no information available about the childhood of the mother and her regression status is unknown. She is cognitively impaired with an IQ of 45. She has attended a special school for children with learning disabilities. Today she lives alone in a sheltered housing unit with daily home care support. She speaks in full meaningful sentences. She is able to read magazines, to write, to knit, to bind shoe lashes and to read the clock. She can manage addition and subtraction of money, but she cannot administer her own finances. Within the frames of known routines, she can manage her personal care, but she cannot initiate or structure activities of daily living or plan and perform simple sequential actions in unfamiliar settings. She is totally dependent on a structured daily schedule. She is easily stressed by anxiety, which occasionally requires medication. There has been no report of seizures. She is overweight with normal height and head circumference. She has a high thoracic kyphosis and a slight lumbar scoliosis. She walks up and down the stairs, but the left foot leads downwards. Her hand muscles are weak and hand movements are ritual. She cannot perform the finger-to-nose-test. Breathing is forced and she breathes only through the mouth. Although she has subtle Rett-like symptoms, she does not meet the required criteria for RTT [1,21].

Patient II:1 is the oldest daughter. At 17 months of age her development corresponded to 12-14 months and her head circumference was below the 1st percentile. She was able to crawl, to walk with slight support and used her hands with pincer grasp. At 2.5 years of age she was removed to foster care for 5 years. During this period she developed stereotypic hand movements and some regression was noticed. Since 7 years of age she has been living in institutions for mentally handicapped individuals. She has preserved some hand function, despite her stereotypic hand movements. She has kyphosis. She walks only a few steps without support, but walks steadily with support. She speaks no words, but knows the sign for coffee and can use eye pointing. She has normal height but she is overweight. She has been treated with antiepileptic drugs for many years, but she is now free of seizures, without medication. She has a normal breathing pattern. She meets all the required and one of the supportive criteria for classical RTT [1,21].

Patient II:2 the younger daughter, was placed in an institution for infants immediately after birth. At 5 months of age she was placed in foster care and she still lives with the same family. At that time she smiled and kept eye contact, but she was passive. She learned to sit at age 8 months and to crawl at age 15 months. She developed a sort of pincer grasp one year old and babbled a lot. At this age her head circumference was below the 2nd percentile. At age 20 months she started kindergarten and some regression was noticed, but she had almost full recovery afterwards. At age 2.5 years she started walking independently and had a vocabulary with small sentences. Since 4 years of age she has kept her hands together in front of her, and since 7 years of age she has been treated for epileptic seizures. At the age of 12 years scoliosis was registered and she underwent surgery 3 years later. At present she is able to sit down, and get up from sitting and lying positions. She can walk independently with small steps, but she is more stable when pushing a wheel chair. She can walk upstairs with support from the banister, but she needs support on the other side while going downstairs. If in mood she can take off her jacket. She has hand dyspraxia. She is growth retarded with microcephaly. She speaks with a high pitch voice and makes sentences up to 5 words. She understands simple messages, but reacts with a long latency. She meets the necessary criteria for atypical RTT [1,21].

### Family B

Family B, (Figure 1B), consists of a mother (patient I:1) and her four children (including patient, II:2, II:3, II:4).

Patient I:1 is the mother of 4 children, from two marriages. Neither of her husbands' had neurological handicaps or MR. One of the two daughters (II:2) from her first marriage is affected, while the other is normal. From the second marriage both children (patient II:3 and II:4) are affected. There is no information about her developmental profile in childhood and her regression status is unknown. She has mild cognitive impairment (IQ: 50-70), but she lives alone without any social support. She has a normal expressive language and she takes care of herself in daily living. She can read magazines and can repeat what she has read. She handles her own money and does her own shopping. However, she does not have any deeper economic insight. She speaks meaningful words and sentences. She is characterized as clumsy. Her muscle tonus is normal and there are no abnormal movements of the hands. Her breathing is forced. No seizures or psychiatric symptoms have been reported. Height, weight and head circumference are normal. She does not have scoliosis. She does not meet the required criteria for RTT [1,21].

Female II:1 is the oldest daughter. There is no information available, but apparently she has a normal phenotype.

Patient II:2, is the oldest affected daughter. She has not been clinically examined by any of the authors and the sparse clinical information is derived from the medical records. Her cognitive impairment and deterioration of the motor function was first noticed in kindergarten. Later full recovery was observed. In primary school, she was diagnosed with MR due to cognitive dysfunction and marked learning disability and received comprehensive educational support. She has ataxia but she can walk without support. Her muscle tonus is normal, but she is clumsy. She has mild thoracic kyphosis. She has mild hand dyspraxia and a tendency to ritual hand movements. She also has tendency to teeth grinding and her breathing is forced. She can speak in short meaningful sentences, however her understanding is limited. No seizures have been reported. She has an intensive eye contact and delayed nociception. She fulfils the necessary criteria for atypical RTT [1,21].

Patient II:3 is the youngest family member. During the first years of life no developmental problems were noted. When she started school a mild cognitive dysfunction with learning disability became evident. Today she is still attending the ordinary school system, but receives special educational support. She speaks in full meaningful sentences, and understands simple messages. She is dependent of known routines and can manage her own personal care. She has no epilepsy and no clinical gastrointestinal symptoms. Her growth has always been normal. Her head circumference is normal. She has a normal muscle tone, with no signs of focal neurological deficits. Her motor abilities are not well coordinated, especially her fine motor skills. There are no pathological orthopaedic signs and no stereotypies. Her breathing is somewhat forced. She does not meet any of the required criteria for RTT [1,21].

Patient II:4 is a boy. He was born to term with normal weight, height and head circumference. His development was apparently normal during the first months, but stagnated at 7-8 months of age. At age 12 months delayed motor development was noticed. He could not sit without support, but he could raise his upper body by support of his arms when lying in prone position. He had general hypotonia as well as marked truncal and fine motor ataxia. He was notably happy and made good emotional contact. At age 20 months he vocalized "mama" and "papa", he was able to sit without support, could receive and throw a ball, and finger feed himself. He had inappropriate screaming spells. The ataxia and hypotonia gradually became more prominent. The head circumference was below the 2.5nd percentile. At this age he started having frequent seizures and received antiepileptic medication. Overall, there was no valid information about regression. CT and MRI scans of the brain and metabolic investigations of organic acids, amino acidopathies and lysosomal disorders as well as lactate and pyruvate in blood and cerebrospinal fluid were normal. He had normal karyotype and genetic test for Angelman syndrome was negative. When examined at age 6 years, his development had stagnated since age 3. He still did not stand or walk alone, but no loss of acquired motor skills had been observed. He seemed less active than before. He had a daily vocabulary of about 5 words, which he used appropriately. Today he has marked motor delay with hypotonia, pronounced ataxia and epilepsy. During the last 2-3 years he has developed hand wringing and dyspraxia. Furthermore, he has some respiratory irregularities, marked teeth grinding and he is growth retarded. Today he presents most of the clinical signs of RTT[1,21].

## Methods

### Clinical evaluation

The patients have been clinically evaluated according to the consensus RTT criteria described in 2001 [1], in order to compare their clinical profiles with previously published family members with RTT and further to the current revised diagnostic criteria from 2010 [21].

### Mutation detection and X inactivation studies

The entire coding *MECP2 *and the flanking intronic sequences were sequenced as described earlier [22,23].

XCI studies were performed using DNA extracted from peripheral blood leukocytes by investigation of the methylation status of the highly polymorphic X-linked androgen receptor (*AR*) locus [24]. The XCI pattern was defined as skewed when the same X chromosome was preferentially inactivated in more than 80% of the cells.

### Determination of the parental origin *de novo MECP2 *frameshift mutations

We selected 24 presumably sporadic RTT patients with a known *MECP2 *frameshift mutation caused by either single-nucleotide deletion/insertion or small intragenic rearrangement. The introns and the 3' untranslated region (3'-UTR) of *MECP2 *were sequenced to identify single-nucleotide-polymorphisms (SNPs). Nine cases were heterozygous for 6 different SNPs localized within introns 2, 3 and 3' UTR (Table 1). Allele-specific PCR was possible in 7 cases and haplotyping of the normal alleles was performed by direct sequencing. In two cases cloning was necessary due to the location of the mutation. A PCR-fragment spanning both the mutation and the SNP was cloned into pGEM-T Esay Vector (Promega) and selected clones were sequenced. By genotyping the respective SNPs in the parents, parent-of-origin was determined.

**Table 1:** The Parental origin of *de novo MECP2 *frameshift mutations

Case no	Age of mother^a^ (Years)	Age of father^a^ (Years)	*MECP2* **mutation **^b^	Intergenic **SNP **^b^	Parental origin
RTT22	31	32	c.215delC	c.1461+1737G > A	Maternal
RTT55	40	43	c.32_50delins11	c.1461+878G > C	Paternal
RTT27	21	25	c. 766_779dup14	c.378-916A > G	Paternal
RTT12	19	30	c.808delC	c.378+266C > T	Maternal
RTT1	29	24	c.1150_1187del38	c.378+648A > G	Paternal
RTT35	30	32	c.1156_1199del44	c.378+266C > T	Paternal
RTT16	26	28	c. 1157_1197del41	c.1461+1737G > A	Paternal
RTT31	24	28	c.1164_1204del44	c.378+266C > T	Paternal
RTT8	23	27	c.1168_1196del29	c.378+266C > T	Paternal

^a ^The parents' age at the time of the RTT patient's birth

^b ^All nucleotides were numbered according to NCBI Reference Sequence: NM_004992.3

## Results

### Family A

Sequence analysis of the *MECP2 *gene in the mother and her two daughters revealed a deletion of 41 basepairs (c.1157_1197del41), within the DPR in exon 4 (Figure 1C). This mutation is predicted to result in a frameshift at codon 386 and subsequent premature truncation of the MeCP2 protein five amino acids downstream (p.L386fs). Results from the *AR*-assay indicated a skewed XCI pattern in the mother (Family A, I:1), whereas both daughters had random XCI pattern (II:1, II:2) (Table 2).

**Table 2:** The X chromosome inactivation patterns obtained from *AR *assays

*AR *assay			
**Family A, I:1**	A270:A273 (12:88)	**Family B, I:1**	A271:A277 (51:49)
**Family A, II:1**	A270:A273 (56:44)	**Family B, II:2**	A274:A277 (46:54)
**Family A, II:2**	A270:A273 (63:37)	**Family B, II:3**	A269:A271 (20:80)

The alleles (A) are indicated with the size of the PCR fragments in base pair. The allele sizes in Family B, indicates the presence of a recombination event. Note that the two half-sisters in family B, does not have the same father.

### Family B

The two maternal half-sisters and the brother had a novel *MECP2 *frameshift mutation, c.1159_delCCinsT, located in the DPR (Figure 1D). This mutation is predicted to result in addition of 21 amino acids followed by a stop codon (p.P387fs). XCI studies showed random XCI patterns in the mother (Family B, I:1) and her oldest affected daughter (II:2), while the second affected daughter had a skewed XCI pattern (II:3) (Table 2).

### Parental origin of the *de novo MECP2 *frameshift mutations

The parental origins were determined in 9 cases with frameshift mutations (Table 1). All five C-terminal frameshift deletions and two mutations generated by small intragenic rearrangements (c.32_50delins11 and c.766_779dup14) were of paternal origin. Two mutations with a deletion of a single cytosine base (c.215delC and c.808delC) were of maternal origin. The mothers' of these two patients did not carry the respective mutations. The parents' ages at the time of the patients' birth are listed in Table 1.

## Discussion

Here we report two new families with frameshift mutations located in the DPR of the *MECP2*. The phenotypes of the affected individuals range from classical RTT to milder cognitive impairment with subtle Rett-like symptoms. Thus, some of these family members represent the clinical link between RTT and asymptomatic carriers of a *MECP2 *mutation. In general, the frameshift mutations in the DPR are commonly reported in RTT patients [19,20]. These patients often fulfil the clinical criteria for classical RTT or a variant form, although few cases have been reported with very mild clinical features [25]. In the extremely mild cases, like some of the present cases (family A, patient I:1 and family B, patient II:3), the detection of a known *MECP2 *mutation is often required to identify the subtle clinical signs as being RTT-like symptoms. Although no clear correlation is demonstrated between genotype, phenotype and XCI in sporadic RTT patients, it seems that a correlation exists in families with RTT females, where an *MECP2 *mutation is segregating (Table 3). We made a similar observation in family A, where the mother had a skewed XCI, most likely in favour of the normal allele, which could explain her mild cognitive impairment with subtle Rett-like symptoms, whereas both her daughters had random XCI pattern and were diagnosed with RTT. In family B, the mother who was an obligate carrier did not want to be tested for the mutation. She had random XCI, which was not in accordance with her obligate carrier status or her mild phenotype. A plausible explanation is that the XCI pattern observed in her blood is not representative for the critical tissue affected in RTT. Alternatively, she could be mosaic for the mutation. Another explanation, which cannot be ruled out without a mutation analysis, is that she has gonadal mosaicism and her mild retardation is due to other factors. The XCI patterns of the two half-sisters were in accordance with their different clinical presentations as the youngest sister with extremely mild symptoms had skewed XCI. The phenotype of the boy has changed with time and at present he meets most of the criteria for a variant form of RTT. However, he has a milder phenotype than expected and does not have neonatal encephalopathy, which has been reported in male with C-terminal deletions in DPR [26]. The large intrafamilial clinical variability observed in family B, suggests the presence of modifying genetic or epigenetic factors, other than XCI.

**Table 3:** Families with RTT in females and *MECP2 *mutation carrier females without RTT

*MECP2 *Mutation	Phenotype male	Phenotype Female	Phenotype Mother	Rf
**Families with RTT in females**				

**p.R106W **c.316C > T		**Two half sisters **Classic RTT	Asympt. mutation negative	[15]
**p.T158M **c.473C > T	Congenital encephalopathy	Classic RTT	Asympt. mutation carrier, SXCI	[14]
**p.R133C **c.397C > T	RTT variant	Classic RTT	Mild MR, mutation carrier, SXCI	[13]
**p.S134C **c.401C > G	RTT variant	RTT variant, random XCI	Asympt. mutation carrier, SXCI	[11]
**P.R168X **c.502C > T		**Two sisters **Classic RTT	Asympt. mutation carrier, SXCI	[16]
**p.V288X **c.806delG	Congenital encephalopathy	**Three females **Mother; mild MR, SXCI Maternal sister; classic RTT Daughter; classic RTT	Grandmother; Asympt., mutation negative, random XCI	[16]
**p.G163fs **c.488_489del	Congenital encephalopathy	Classic RTT	Asympt. mutation negative	[12]
**p.G252fs **c.754insC	Congenital encephalopathy	Classic RTT	Asympt. mutation negative	[17]
**p.L386fs **c.1157_1197del41		**Two sisters **Classic RTT, random XCI RTT Variant, random XCI	Mild MR, mutation carrier, SXCI	**Present case**
**p.P387fs **c.1159_delCCinsT	RTT variant	**Two sisters **RTT Variant, random XCI Mild MR, SXCI	Mild MR, mutation?, random XCI	**Present case**
**p.P388fs **c.1164_1207del44	**Two males **Uncle and nephew RTT variants	**Two females **Mother mild MR, SXCI Daughter classic RTT	Grandmother, mild MR, mutation carrier, SXCI	[10]
**g.58483_65650del;** **g.65664_66958del**	Congenital encephalopathy	Classic RTT	Mild MR, mutation carrier, SXCI	[30]

***MECP2 *mutation carrier females without RTT**				

**p.T158M **c.473C > T	**Two brothers **Congenital encephalopathy		Asympt. mutation carrier, SXCI	[31]
**p.P322S **c.964C > T	RTT variant		Mild MR, mutation carrier, random XCI	[32]
**p.P380fs **c.1140del86	**Two brothers **MR, neurologic symptoms, dimorphic features		Asympt. mutation carrier, XCI not available	[28]
**p.P384fs **c.1154del32	Congenital encephalopathy		Asympt. mutation carrier, SXCI	[26]
**p.L386fs **c.1158del44	RTT variant		Asympt. mutation carrier, SXCI	[27]

MR, presented with mental retardation; SXCI, Skewed X chromosome inactivation

All nucleotides were numbered according to NCBI Reference Sequence: NM_004992.3

Four families with deletions in the DPR have previously been reported [10,26-28]. In three of these families the mothers were asymptomatic carriers with skewed XCI pattern presumably in favour of the normal allele (Table 3). The fourth family included three generations (3 females and 2 males) with a 1164_1207del44 mutation [10]. The grandmother had learning disability, the mother had cognitive delay, whereas the granddaughter had classical RTT. Both males presented with RTT-like features including deceleration of head growth and transient presence of stereotypic hand movements. The XCI patterns obtained for each of the female family members correlated with their respective phenotypes (Table 3).

The present study suggests that there may be a group of unrecognized asymptomatic or mildly affected mothers carrying a *MECP2 *frameshift mutation. Like the present families, these carrier mothers may not be recognized until they give birth to children with RTT. As summarized in table 3 more than half of the families with RTT have a frameshift mutation, and a clear correlation exists between the XCI profile in blood and the phenotypes of the respective family members. It is known from cases with sporadic RTT, that over 70% have the C > T transition, (RettBASE, http://mecp2.chw.edu.au), which are almost exclusively of paternal origin [4-7] and the blood XCI profiles do not correlate with their phenotypes [29]. Therefore another segregation mechanism may exist for *MECP2 *frameshift mutations, which can influence a more favourable phenotype, e.g. XCI, which could explain the over-representation of frameshift mutations in the families with RTT. It was not possible to determine the parental origin of the mutations in our families. As the parental origin of *de novo *frameshift *MECP2 *mutations are only scarcely reported, we attempted to determine parental origin of *de novo *frameshift mutations in our RTT cohort. Out of 24 sporadic RTT cases with frameshift mutations we could determine the parental origin in 9. Surprisingly, we found that the majority of the *de novo *frameshift mutations occurred on the paternally derived X chromosome (7/9 cases) without paternal age effect. All five cases with C-terminal deletions were of paternal origin, which suggests that the primary mutational event in family A had occurred on the mother's paternal X chromosome. The two mutations with maternal origin were both deletions of a single cytosine (c.215delC and c. 808delC) predicted to lead to early premature truncation of the MeCP2. The mothers' of these patients were both negative for the respective mutations. Whether single base pair deletions are more frequently maternally derived is yet to be investigated. In two previously reported cases with sporadic RTT, the frameshift mutations (c.806delG and c.677insA) occurred on the maternally derived X chromosome [5,7]. Together these results indicate that frameshift mutations involving only a single base pair have a tendency to be of maternal origin than first anticipated, but as listed in Table 3 the familial mutations are not predominated by single base pair deletions or insertions. The underlying mechanisms for the sex bias among *de novo MECP2 *mutations in RTT patients require further investigation, but the sparse number of reported males with a pathogenic *MECP2 *mutation and families with RTT seem to support ours and others findings of paternal origin [4-7]. Furthermore, these findings also indicate that the existence of a large number of unknown *MECP2 *carriers is probably unlikely.

The recurrence risk of RTT is low, but an empirical estimation should be taken with caution. Based on the Danish and the Norwegian RTT populations with identified *MECP2 *mutations, the incidence of the RTT families compared to the sporadic cases is approximately 1:150 (unpublished data). As investigation of the mothers' XCI status cannot provide sufficient evidence to exclude a carrier status due to mosaicism, determination of the paternal origin of the *MECP2 *mutation, when possible, may give further information on whether a new RTT case is sporadic. The knowledge of rare mutation events described in the present families underlines importance of genetic counselling and discussion of the possibility of prenatal diagnosis in families with a proband who has a *MECP2 *mutation.

## Abbreviations

RTT: Rett syndrome; *MECP2*: Methyl-CpG-binding protein 2 (gene); XCI: X chromosome inactivation; *AR*:Androgen receptor (gene); C:Cytosine; T:Tymine; DPR:Deleted prone region; MR: Mental retardation, 3' UTR: 3' untranslated region

## Competing interests

The authors declare that they have no competing interests.

## Authors' contributions

KR drafted the manuscript and was responsible for the molecular genetic studies and interpretation of data. GR, KF made a substantial contribution to evaluate the clinical data and finalizing the manuscript and ZT with finalizing the manuscript. MD, KLE contributed with genetic diagnostic and interpretation of data. JBN, OHS with clinical information, samples and patient descriptions. All authors read and approved the final manuscript.
